# Crystal structure of tebipenem pivoxil

**DOI:** 10.1107/S2056989018010770

**Published:** 2018-08-10

**Authors:** Chao Tang, Li Cai, Shuai Liu, Zhiwei Zheng, Gen Li, Jianli Chen, Qiang Sui

**Affiliations:** a China State Institute of Pharmaceutical Industry, 285 Gebaini Rd, Shanghai 201203, People’s Republic of China; b University of South Carolina Lancaster, 476 Hubbard Drive, Lancaster, SC 29720, USA

**Keywords:** crystal structure, carbapenem, anti­biotics, tebipenem, prodrug, hydrogen bonding

## Abstract

The mol­ecular structure of the first orally active carbapenem anti­bacterial agent, tebipenem pivoxil (systematic name: (2,2-di­methyl­propano­yloxy)methyl (4*R*,5*S*,6*S*)-3-{[1-(4,5-di­hydro-1,3-thia­zol-2-yl)azetidin-3-yl]sulfanyl}-6-[(1*R*)-1-hy­droxy­eth­yl]-4-methyl-7-oxo-1-aza­bicyclo­[3.2.0]hept-2-ene-2-carboxyl­ate), C_22_H_31_N_3_O_6_S_2_, has been determined and the configurations of the four chiral centers validated. The title compound crystallizes in the triclinic space group *P*1 with one mol­ecule in the unit cell. Three out of the four rings adopt planar conformations while the thia­zolinyl ring adopts an enveloped conformation. In the crystal, O—H⋯N hydrogen bonds link the mol­ecules into chains along [10].

## Abstract 2

The mol­ecular structure of the first orally active carbapenem agent tebipenem pivoxil is described.

## Chemical context   

Carbapenem anti­biotics, like all *β*-lactam anti­bacterials that bind to and inhibit the peptidoglycan cross-linking transpeptidases, have attracted increasing attention recently because of their broader spectrum activities and stronger bactericidal actions compared to cephalosporins and penicillins. Since the first carbapenem structure thienamycin, a natural product derived from *Streptomyces cattleya*, was isolated in 1976 (Johnston *et al.*, 1978 ▸), a handful of subsequent parenteral carbapenem agents, such as imipenem, panipenem, meropenem, biapenem, have been developed based on this parent compound and used clinically for the treatment of severe bacterial infections.
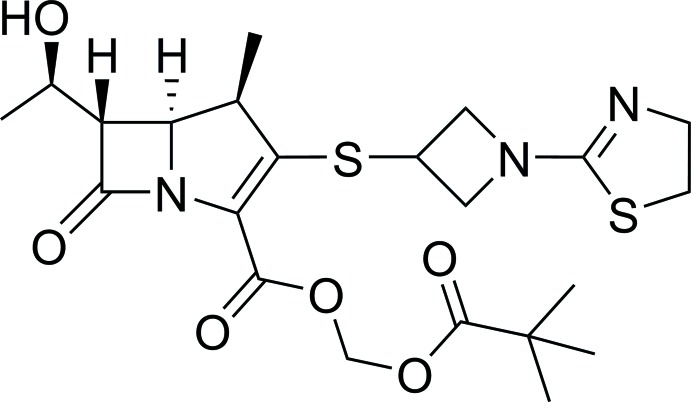


Tebipenem pivoxil (see scheme[Chem scheme1]), as a novel oral carbapenem agent, was approved by the Pharmaceuticals and Medical Devices Agency of Japan (PMDA) on Apr 22, 2009. It was developed and marketed as Orapenem® by Meiji Seika in Japan (as of 05/16/2016, the only approved country/area for its usage was Japan for treating children, as these oral anti­biotics are often better tolerated than infusions) (Kijima *et al.*, 2009 ▸). It is a prodrug that is quickly hydrolysed to the active anti­microbial agent LJC11,036 (**5**, reaction scheme[Chem scheme2]) because the absorption rate of the pivaloyloxymethyl ester is higher than that of other prodrug-type *β*-lactam anti­biotics (Kato *et al.*, 2010 ▸). The active metabolite **5** shows potent and well-balanced anti­bacterial activity and also shows higher stability to human renal de­hydro­peptidase-I than meropenem (Isoda *et al.*, 2006*a* ▸; Kobayashi *et al.*, 2005 ▸). Research has also revealed that the tebipenbem ac­yl–*β*-lactamase covalent complex remains very stable for longer than 90 min, partly explaining its resistance towards hydrolysis (Papp-Wallace *et al.*, 2011 ▸).
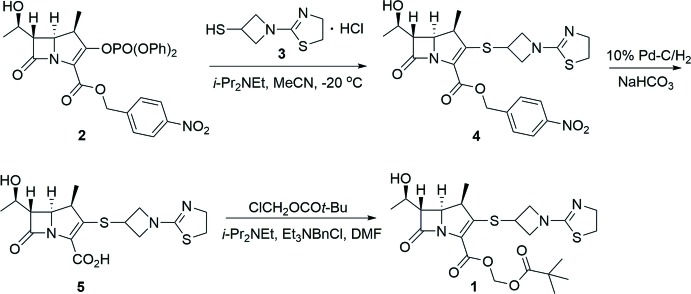


Tebipenem pivoxil has a complex structure with four chiral centers and a 1-(1,3-thia­zolin-2-yl)azetidin-3-yl­thio side chain at the C-2 position. We hope the structural elucidation will facilitate future mechanistic studies of this mol­ecule and of its inter­actions with enzymes that are responsible for bacterial resistance.

## Structural commentary   

**Figure 1:**
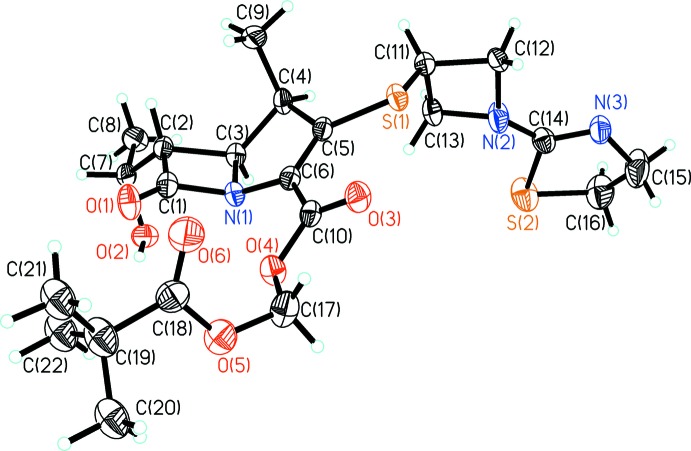
The mol­ecular structure of the title compound, showing the atom labelling and 30% probability displacement ellipsoids.

Tebipenem pivoxil (Fig. 1 ▸) crystallizes in the triclinic space group *P*1 with one mol­ecule in the unit cell. The present crystal structure dertermination allowed the configurations of the four chiral centers to be validated as: C2*S*, C3*S*, C4*R*, C7*R*. Rings I (N1/C1–C3), II (N1/C3–C6) and III (N2/C11–C13) adopt planar conformations (with r.m.s. deviations of 0.0251, 0.0838, and 0.0967 Å, respectively) while ring IV (N3/S2/C14–C16) adopts an envelope conformation with atom C16 as the flap. The dihedral angles between rings I and II, II and III, and III and IV are 46.7 (2), 85.7 (2), and 11.9 (4)°, respectively. Atoms C9 (meth­yl) and C7 are located above and below the planes of rings I and II because of steric hindrance.

## Supra­molecular features   

**Figure 2:**
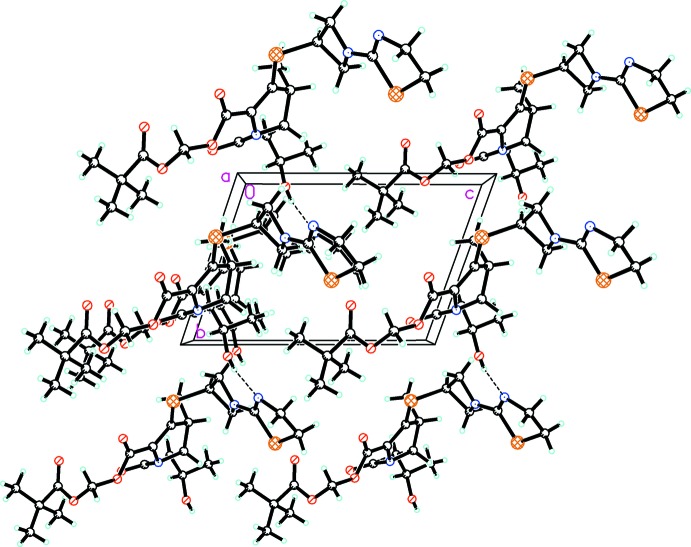
The crystal packing viewed along the crystallographic *a* axis showing the O—H⋯N hydrogen bonds (Table 1 ▸) as dashed lines.

In the crystal, O—H⋯N hydrogen bonds (Table 1 ▸) link the mol­ecules into chains along [1
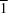
0]. C—H⋯O hydrogen bonds are also observed. The packing viewed along the *a* axis is shown in Fig. 2 ▸.

## Database survey   

The tebipenem pivoxil we obtained was well characterized spectroscopically and carefully compared with reference values (Isoda *et al.*, 2006*a* ▸). To the best of our knowledge, including a search of the Cambridge Structural Database (CSD Version 5.39; Groom *et al.*, 2016 ▸), no single crystal structure determination has previously been reported for this drug.

## Synthesis and crystallization   

As shown in the reaction scheme[Chem scheme2] (also see Supporting Information), 3-mercapto-1-(1,3-thia­zolin-2-yl)-azetidine hydro­chloride (**3**) was first synthesized according to a method previously reported (Isoda *et al.*, 2006*b* ▸) with minor optimizations. The side chain **3** was then coupled with the commercially available carbapenem core (**2**), followed by hydrogenation/deprotection and SN_2_ esterification to afford the desired tebipenem pivoxil **1** (Isoda *et al.*, 2006*a* ▸,*b* ▸). Instead of using column chromatography, we successfully obtained pure tebipenem pivoxil on a relatively large scale through recrystallization from ethyl acetate, yielding colourless block-shaped crystals. The HPLC spectrum of the final product showed a single peak with less than 0.1% of impurities. [α]_D_^8^ = +9.6°, m.p. = 407–409 K. Elemental analysis calculated for C_22_H_31_N_3_O_6_S_2_: C, 53.10; H, 6.28; N, 8.44; S, 12.89; Found: C, 53.13; H, 6.32; N, 8.45; S, 12.94. HRESI–MS calculated for C_22_H_32_N_3_O_6_S_2_ ([*M* + H]^+^): 498.1727, found: 498.1867. The structure has also been characterized with ^1^H NMR, ^13^C NMR, and IR spectroscopy. ^1^H NMR, ^13^C NMR, and IR spectra of tebipenem pivoxil **1** are included in the supporting information and compared with reference values, including the assignment of NMR chemical shifts and IR absorption bands (Isoda *et al.*, 2006*a* ▸).

## Refinement   

Crystal data, data collection and structure refinement details are summarized in Table 2 ▸. In the refinement, all H atoms were positioned geometrically and refined as riding: C—H = 0.96–0.98 Å with *U*_iso_(H) = 1.2*U*_eq_(C) or 1.5*U*_eq_(C-meth­yl).

## Supplementary Material

Crystal structure: contains datablock(s) I. DOI: 10.1107/S2056989018010770/ex2010sup1.cif

Structure factors: contains datablock(s) I. DOI: 10.1107/S2056989018010770/ex2010Isup2.hkl

Synthetic conditions and 1H NMR, 13C NMR, and IR spectra of tebipenem pivoxil.. DOI: 10.1107/S2056989018010770/ex2010sup3.pdf

Supporting information file. DOI: 10.1107/S2056989018010770/ex2010Isup4.cml

CCDC reference: 1816052

Additional supporting information:  crystallographic information; 3D view; checkCIF report

## Figures and Tables

**Table 1:** Hydrogen-bond geometry (Å, °)

*D*—H⋯*A*	*D*—H	H⋯*A*	*D*⋯*A*	*D*—H⋯*A*
O2—H2*A*⋯N3^i^	0.82	2.01	2.816 (6)	169
C11—H11*A*⋯O2^ii^	0.98	2.43	3.366 (6)	160

Symmetry codes: (i) 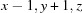; (ii) 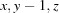.

**Table 2:** Experimental details

Crystal data	
Chemical formula	C_22_H_31_N_3_O_6_S_2_
*M*_r_	497.62
Crystal system, space group	Triclinic, *P*1
Temperature (K)	296
*a*, *b*, *c* (Å)	7.7292 (10), 7.9892 (9), 11.2035 (13)
α, β, γ (°)	108.300 (7), 92.553 (7), 101.499 (8)
*V* (Å^3^)	639.36 (14)
*Z*	1
Radiation type	Cu *K*α
μ (mm^−1^)	2.23
Crystal size (mm)	0.17 × 0.12 × 0.10

Data collection	
Diffractometer	Bruker APEXII CCD
Absorption correction	Multi-scan (*SADABS*; Bruker, 2014 ▸)
*T*_min_, *T*_max_	0.703, 0.808
No. of measured, independent and observed [*I* > 2σ(*I*)] reflections	3454, 2483, 2389
*R*_int_	0.019
(sin θ/λ)_max_ (Å^−1^)	0.592

Refinement	
*R*[*F* ^2^ > 2σ(*F* ^2^)], *wR*(*F* ^2^), *S*	0.042, 0.115, 1.04
No. of reflections	2483
No. of parameters	298
No. of restraints	3
H-atom treatment	H-atom parameters constrained
Δρ_max_, Δρ_min_ (e Å^−3^)	0.33, −0.21
Absolute structure	Flack *x* determined using 531 quotients [(*I* ^+^)−(*I* ^−^)]/[(*I* ^+^)+(*I* ^−^)] (Parsons *et al.*, 2013 ▸)
Absolute structure parameter	0.140 (12)

Computer programs: *APEX2* and *SAINT* (Bruker, 2014 ▸), *SHELXS2018/3* and *SHELXTL* (Sheldrick, 2008 ▸) and *SHELXL2018/3* (Sheldrick, 2015 ▸).

## supplementary crystallographic information

### Crystal data

**Table:**

C_22_H_31_N_3_O_6_S_2_	*Z* = 1
*M**_r_* = 497.62	*F*(000) = 264
Triclinic, *P*1	*D*_x_ = 1.292 Mg m^−^^3^
*a* = 7.7292 (10) Å	Cu *K*α radiation, λ = 1.54178 Å
*b* = 7.9892 (9) Å	Cell parameters from 2598 reflections
*c* = 11.2035 (13) Å	θ = 4.2–65.6°
α = 108.300 (7)°	µ = 2.23 mm^−^^1^
β = 92.553 (7)°	*T* = 296 K
γ = 101.499 (8)°	Block, colorless
*V* = 639.36 (14) Å^3^	0.17 × 0.12 × 0.10 mm

### Data collection

**Table:**

Bruker APEXII CCD diffractometer	2389 reflections with *I* > 2σ(*I*)
φ and ω scans	*R*_int_ = 0.019
Absorption correction: multi-scan (SADABS; Bruker, 2014)	θ_max_ = 65.9°, θ_min_ = 4.2°
*T*_min_ = 0.703, *T*_max_ = 0.808	*h* = −7→9
3454 measured reflections	*k* = −9→9
2483 independent reflections	*l* = −13→12

### Refinement

**Table:**

Refinement on *F*^2^	Hydrogen site location: inferred from neighbouring sites
Least-squares matrix: full	H-atom parameters constrained
*R*[*F*^2^ > 2σ(*F*^2^)] = 0.042	*w* = 1/[σ^2^(*F*_o_^2^) + (0.0826*P*)^2^ + 0.0878*P*] where *P* = (*F*_o_^2^ + 2*F*_c_^2^)/3
*wR*(*F*^2^) = 0.115	(Δ/σ)_max_ = 0.015
*S* = 1.04	Δρ_max_ = 0.33 e Å^−^^3^
2483 reflections	Δρ_min_ = −0.21 e Å^−^^3^
298 parameters	Absolute structure: Flack *x* determined using 531 quotients [(*I*^+^)-(*I*^-^)]/[(*I*^+^)+(*I*^-^)] (Parsons *et al.*, 2013)
3 restraints	Absolute structure parameter: 0.140 (12)

### Special details

**Table:**

Geometry. All esds (except the esd in the dihedral angle between two l.s. planes) are estimated using the full covariance matrix. The cell esds are taken into account individually in the estimation of esds in distances, angles and torsion angles; correlations between esds in cell parameters are only used when they are defined by crystal symmetry. An approximate (isotropic) treatment of cell esds is used for estimating esds involving l.s. planes.

### Fractional atomic coordinates and isotropic or equivalent isotropic displacement parameters (Å^2^)

**Table:**

	*x*	*y*	*z*	*U*_iso_*/*U*_eq_	
S1	0.33764 (12)	0.37913 (11)	0.01567 (9)	0.0531 (3)	
S2	0.6657 (3)	0.6243 (2)	0.50488 (14)	0.0889 (5)	
O1	−0.0384 (5)	0.8665 (5)	−0.1269 (3)	0.0695 (9)	
O2	−0.0964 (4)	1.0714 (4)	0.1973 (3)	0.0601 (8)	
H2A	−0.139807	1.152553	0.240566	0.090*	
O3	0.4728 (4)	0.6066 (5)	−0.1353 (3)	0.0626 (8)	
O4	0.3985 (4)	0.8733 (4)	−0.1081 (3)	0.0589 (8)	
O5	0.4493 (5)	1.0081 (6)	−0.2577 (4)	0.0781 (11)	
O6	0.2673 (7)	0.7659 (6)	−0.3881 (6)	0.1060 (16)	
N1	0.1226 (4)	0.7928 (4)	0.0285 (3)	0.0447 (7)	
N2	0.5213 (7)	0.3896 (6)	0.2801 (4)	0.0737 (12)	
N3	0.7629 (6)	0.3330 (6)	0.3725 (4)	0.0679 (11)	
C1	−0.0250 (6)	0.8214 (6)	−0.0352 (4)	0.0486 (9)	
C2	−0.1483 (5)	0.7724 (5)	0.0574 (4)	0.0455 (8)	
H2B	−0.238265	0.661201	0.014328	0.055*	
C3	0.0144 (5)	0.7296 (5)	0.1179 (4)	0.0442 (8)	
H3B	0.050788	0.808606	0.205772	0.053*	
C4	0.0397 (6)	0.5350 (6)	0.0968 (4)	0.0498 (9)	
H4A	0.058878	0.516830	0.178516	0.060*	
C5	0.2114 (5)	0.5408 (5)	0.0341 (4)	0.0437 (8)	
C6	0.2416 (5)	0.6785 (5)	−0.0123 (4)	0.0436 (8)	
C7	−0.2320 (5)	0.9153 (5)	0.1391 (4)	0.0483 (9)	
H7A	−0.316888	0.944012	0.085209	0.058*	
C8	−0.3299 (7)	0.8508 (7)	0.2361 (5)	0.0651 (12)	
H8A	−0.421147	0.744567	0.193571	0.098*	
H8B	−0.247930	0.822482	0.289674	0.098*	
H8C	−0.382809	0.944259	0.286473	0.098*	
C9	−0.1081 (6)	0.3822 (7)	0.0108 (7)	0.0762 (16)	
H9A	−0.215396	0.379378	0.050815	0.114*	
H9B	−0.127753	0.402322	−0.068287	0.114*	
H9C	−0.073970	0.268830	−0.004165	0.114*	
C10	0.3811 (5)	0.7099 (6)	−0.0923 (4)	0.0472 (9)	
C11	0.2935 (6)	0.3012 (6)	0.1482 (5)	0.0535 (10)	
H11A	0.175564	0.222769	0.139734	0.064*	
C12	0.4501 (6)	0.2200 (5)	0.1758 (5)	0.0532 (10)	
H12A	0.415244	0.115292	0.202918	0.064*	
H12B	0.525236	0.196247	0.108146	0.064*	
C13	0.3520 (6)	0.4482 (6)	0.2791 (5)	0.0581 (11)	
H13A	0.364717	0.570857	0.277752	0.070*	
H13B	0.280849	0.428426	0.344663	0.070*	
C14	0.6465 (6)	0.4232 (6)	0.3738 (4)	0.0559 (11)	
C15	0.8971 (11)	0.4207 (12)	0.4850 (8)	0.116 (3)	
H15A	1.004355	0.482093	0.461013	0.140*	
H15B	0.927124	0.329086	0.516839	0.140*	
C16	0.8293 (11)	0.5528 (11)	0.5860 (6)	0.098 (2)	
H16A	0.924735	0.655012	0.632517	0.118*	
H16B	0.775573	0.496738	0.644719	0.118*	
C17	0.5186 (7)	0.9092 (9)	−0.1956 (6)	0.0739 (14)	
H17A	0.634318	0.976545	−0.150543	0.089*	
H17B	0.532612	0.796482	−0.255647	0.089*	
C18	0.3224 (8)	0.9266 (8)	−0.3535 (5)	0.0724 (14)	
C19	0.2625 (11)	1.0577 (9)	−0.4111 (5)	0.0854 (18)	
C20	0.406 (2)	1.2163 (17)	−0.4024 (14)	0.181 (6)	
H20A	0.455241	1.276802	−0.315584	0.271*	
H20B	0.497695	1.176572	−0.451723	0.271*	
H20C	0.358495	1.298285	−0.434103	0.271*	
C21	0.179 (2)	0.9556 (19)	−0.5419 (9)	0.183 (6)	
H21A	0.087040	0.855032	−0.541445	0.274*	
H21B	0.127395	1.033571	−0.575774	0.274*	
H21C	0.266596	0.911858	−0.593394	0.274*	
C22	0.1304 (18)	1.1339 (17)	−0.3284 (9)	0.142 (4)	
H22A	0.188241	1.199331	−0.244299	0.213*	
H22B	0.082558	1.214142	−0.361897	0.213*	
H22C	0.035952	1.036984	−0.326218	0.213*	

### Atomic displacement parameters (Å^2^)

**Table:**

	*U*^11^	*U*^22^	*U*^33^	*U*^12^	*U*^13^	*U*^23^
S1	0.0556 (6)	0.0528 (5)	0.0564 (6)	0.0265 (4)	0.0067 (4)	0.0168 (4)
S2	0.1153 (13)	0.0862 (10)	0.0592 (7)	0.0530 (9)	0.0044 (7)	−0.0025 (7)
O1	0.078 (2)	0.096 (3)	0.0542 (19)	0.0412 (19)	0.0077 (16)	0.0382 (18)
O2	0.0553 (17)	0.0497 (16)	0.070 (2)	0.0134 (14)	0.0171 (15)	0.0107 (15)
O3	0.0572 (18)	0.0701 (19)	0.067 (2)	0.0282 (17)	0.0193 (15)	0.0220 (16)
O4	0.0610 (18)	0.0706 (18)	0.0592 (18)	0.0253 (15)	0.0183 (14)	0.0332 (16)
O5	0.081 (2)	0.102 (3)	0.0598 (19)	0.014 (2)	0.0054 (17)	0.042 (2)
O6	0.099 (3)	0.077 (3)	0.126 (4)	0.020 (2)	−0.015 (3)	0.014 (3)
N1	0.0461 (17)	0.0475 (17)	0.0448 (17)	0.0201 (14)	0.0055 (14)	0.0155 (14)
N2	0.072 (3)	0.076 (3)	0.068 (3)	0.045 (2)	−0.005 (2)	0.002 (2)
N3	0.064 (2)	0.065 (2)	0.064 (2)	0.029 (2)	−0.0105 (19)	0.001 (2)
C1	0.050 (2)	0.052 (2)	0.041 (2)	0.0208 (18)	0.0003 (16)	0.0081 (18)
C2	0.0405 (19)	0.048 (2)	0.046 (2)	0.0151 (16)	−0.0009 (16)	0.0099 (17)
C3	0.043 (2)	0.052 (2)	0.0400 (18)	0.0173 (16)	0.0043 (15)	0.0141 (17)
C4	0.044 (2)	0.058 (2)	0.058 (2)	0.0244 (18)	0.0084 (17)	0.026 (2)
C5	0.0399 (18)	0.0476 (19)	0.0424 (19)	0.0142 (16)	−0.0011 (15)	0.0112 (17)
C6	0.0399 (19)	0.047 (2)	0.0416 (18)	0.0131 (16)	0.0002 (14)	0.0097 (16)
C7	0.0414 (19)	0.048 (2)	0.058 (2)	0.0179 (17)	0.0050 (17)	0.0158 (18)
C8	0.062 (3)	0.061 (3)	0.079 (3)	0.019 (2)	0.024 (2)	0.026 (2)
C9	0.045 (2)	0.053 (3)	0.129 (5)	0.008 (2)	0.004 (3)	0.031 (3)
C10	0.0422 (19)	0.057 (2)	0.043 (2)	0.0152 (19)	−0.0019 (16)	0.0155 (18)
C11	0.045 (2)	0.051 (2)	0.070 (3)	0.0151 (18)	0.0032 (19)	0.026 (2)
C12	0.057 (2)	0.043 (2)	0.062 (2)	0.0210 (18)	0.001 (2)	0.0162 (18)
C13	0.059 (3)	0.066 (3)	0.063 (3)	0.035 (2)	0.016 (2)	0.026 (2)
C14	0.063 (3)	0.057 (2)	0.049 (2)	0.025 (2)	0.008 (2)	0.013 (2)
C15	0.098 (5)	0.119 (6)	0.102 (5)	0.055 (5)	−0.043 (5)	−0.016 (5)
C16	0.101 (5)	0.115 (5)	0.067 (4)	0.036 (4)	−0.014 (3)	0.010 (4)
C17	0.056 (3)	0.113 (4)	0.070 (3)	0.024 (3)	0.019 (2)	0.051 (3)
C18	0.069 (3)	0.072 (3)	0.069 (3)	0.012 (3)	0.008 (3)	0.016 (3)
C19	0.128 (5)	0.087 (4)	0.048 (3)	0.037 (4)	0.001 (3)	0.025 (3)
C20	0.247 (16)	0.137 (9)	0.167 (11)	0.007 (9)	−0.005 (11)	0.089 (8)
C21	0.267 (18)	0.196 (12)	0.070 (5)	0.079 (12)	−0.037 (7)	0.014 (6)
C22	0.190 (11)	0.185 (10)	0.095 (6)	0.122 (9)	0.017 (6)	0.057 (6)

### Geometric parameters (Å, º)

**Table:**

S1—C5	1.737 (4)	C8—H8A	0.9600
S1—C11	1.802 (4)	C8—H8B	0.9600
S2—C14	1.778 (5)	C8—H8C	0.9600
S2—C16	1.807 (7)	C9—H9A	0.9600
O1—C1	1.197 (5)	C9—H9B	0.9600
O2—C7	1.409 (5)	C9—H9C	0.9600
O2—H2A	0.8200	C11—C13	1.544 (7)
O3—C10	1.196 (5)	C11—C12	1.546 (6)
O4—C10	1.354 (5)	C11—H11A	0.9800
O4—C17	1.433 (6)	C12—H12A	0.9700
O5—C18	1.323 (7)	C12—H12B	0.9700
O5—C17	1.370 (7)	C13—H13A	0.9700
O6—C18	1.198 (7)	C13—H13B	0.9700
N1—C1	1.413 (5)	C15—C16	1.480 (11)
N1—C6	1.414 (6)	C15—H15A	0.9700
N1—C3	1.477 (5)	C15—H15B	0.9700
N2—C14	1.319 (6)	C16—H16A	0.9700
N2—C12	1.468 (6)	C16—H16B	0.9700
N2—C13	1.476 (6)	C17—H17A	0.9700
N3—C14	1.258 (6)	C17—H17B	0.9700
N3—C15	1.481 (7)	C18—C19	1.523 (8)
C1—C2	1.527 (6)	C19—C21	1.479 (10)
C2—C7	1.500 (6)	C19—C20	1.483 (15)
C2—C3	1.549 (5)	C19—C22	1.499 (13)
C2—H2B	0.9800	C20—H20A	0.9600
C3—C4	1.551 (6)	C20—H20B	0.9600
C3—H3B	0.9800	C20—H20C	0.9600
C4—C9	1.527 (7)	C21—H21A	0.9600
C4—C5	1.528 (6)	C21—H21B	0.9600
C4—H4A	0.9800	C21—H21C	0.9600
C5—C6	1.342 (6)	C22—H22A	0.9600
C6—C10	1.466 (6)	C22—H22B	0.9600
C7—C8	1.512 (6)	C22—H22C	0.9600
C7—H7A	0.9800		
			
C5—S1—C11	102.76 (19)	C13—C11—H11A	114.7
C14—S2—C16	88.8 (3)	C12—C11—H11A	114.7
C7—O2—H2A	109.5	S1—C11—H11A	114.7
C10—O4—C17	116.1 (4)	N2—C12—C11	88.2 (3)
C18—O5—C17	120.2 (5)	N2—C12—H12A	114.0
C1—N1—C6	132.4 (3)	C11—C12—H12A	114.0
C1—N1—C3	93.1 (3)	N2—C12—H12B	114.0
C6—N1—C3	108.6 (3)	C11—C12—H12B	114.0
C14—N2—C12	128.7 (4)	H12A—C12—H12B	111.2
C14—N2—C13	130.5 (5)	N2—C13—C11	88.0 (4)
C12—N2—C13	92.8 (4)	N2—C13—H13A	114.0
C14—N3—C15	111.1 (5)	C11—C13—H13A	114.0
O1—C1—N1	131.4 (4)	N2—C13—H13B	114.0
O1—C1—C2	136.6 (4)	C11—C13—H13B	114.0
N1—C1—C2	91.9 (3)	H13A—C13—H13B	111.2
C7—C2—C1	118.6 (3)	N3—C14—N2	124.9 (5)
C7—C2—C3	117.9 (3)	N3—C14—S2	117.5 (4)
C1—C2—C3	86.1 (3)	N2—C14—S2	117.3 (4)
C7—C2—H2B	110.7	N3—C15—C16	111.0 (6)
C1—C2—H2B	110.7	N3—C15—H15A	109.4
C3—C2—H2B	110.7	C16—C15—H15A	109.4
N1—C3—C2	88.6 (3)	N3—C15—H15B	109.4
N1—C3—C4	104.6 (3)	C16—C15—H15B	109.4
C2—C3—C4	123.7 (3)	H15A—C15—H15B	108.0
N1—C3—H3B	112.2	C15—C16—S2	105.3 (5)
C2—C3—H3B	112.2	C15—C16—H16A	110.7
C4—C3—H3B	112.2	S2—C16—H16A	110.7
C9—C4—C5	109.9 (4)	C15—C16—H16B	110.7
C9—C4—C3	115.8 (4)	S2—C16—H16B	110.7
C5—C4—C3	100.8 (3)	H16A—C16—H16B	108.8
C9—C4—H4A	110.0	O5—C17—O4	108.0 (4)
C5—C4—H4A	110.0	O5—C17—H17A	110.1
C3—C4—H4A	110.0	O4—C17—H17A	110.1
C6—C5—C4	110.6 (4)	O5—C17—H17B	110.1
C6—C5—S1	125.5 (3)	O4—C17—H17B	110.1
C4—C5—S1	123.7 (3)	H17A—C17—H17B	108.4
C5—C6—N1	110.9 (3)	O6—C18—O5	120.9 (6)
C5—C6—C10	125.1 (4)	O6—C18—C19	126.3 (6)
N1—C6—C10	124.0 (3)	O5—C18—C19	112.8 (5)
O2—C7—C2	107.8 (3)	C21—C19—C20	113.4 (9)
O2—C7—C8	111.5 (4)	C21—C19—C22	111.2 (10)
C2—C7—C8	111.3 (4)	C20—C19—C22	105.0 (9)
O2—C7—H7A	108.7	C21—C19—C18	108.6 (7)
C2—C7—H7A	108.7	C20—C19—C18	113.3 (8)
C8—C7—H7A	108.7	C22—C19—C18	105.0 (6)
C7—C8—H8A	109.5	C19—C20—H20A	109.5
C7—C8—H8B	109.5	C19—C20—H20B	109.5
H8A—C8—H8B	109.5	H20A—C20—H20B	109.5
C7—C8—H8C	109.5	C19—C20—H20C	109.5
H8A—C8—H8C	109.5	H20A—C20—H20C	109.5
H8B—C8—H8C	109.5	H20B—C20—H20C	109.5
C4—C9—H9A	109.5	C19—C21—H21A	109.5
C4—C9—H9B	109.5	C19—C21—H21B	109.5
H9A—C9—H9B	109.5	H21A—C21—H21B	109.5
C4—C9—H9C	109.5	C19—C21—H21C	109.5
H9A—C9—H9C	109.5	H21A—C21—H21C	109.5
H9B—C9—H9C	109.5	H21B—C21—H21C	109.5
O3—C10—O4	123.8 (4)	C19—C22—H22A	109.5
O3—C10—C6	124.3 (4)	C19—C22—H22B	109.5
O4—C10—C6	111.9 (4)	H22A—C22—H22B	109.5
C13—C11—C12	87.3 (3)	C19—C22—H22C	109.5
C13—C11—S1	114.7 (3)	H22A—C22—H22C	109.5
C12—C11—S1	107.7 (3)	H22B—C22—H22C	109.5
			
C6—N1—C1—O1	−56.9 (7)	C17—O4—C10—O3	8.5 (6)
C3—N1—C1—O1	−175.8 (5)	C17—O4—C10—C6	−173.7 (4)
C6—N1—C1—C2	122.9 (4)	C5—C6—C10—O3	7.5 (6)
C3—N1—C1—C2	4.0 (3)	N1—C6—C10—O3	−173.8 (4)
O1—C1—C2—C7	−64.2 (7)	C5—C6—C10—O4	−170.2 (4)
N1—C1—C2—C7	116.0 (4)	N1—C6—C10—O4	8.4 (5)
O1—C1—C2—C3	176.0 (6)	C5—S1—C11—C13	−61.7 (3)
N1—C1—C2—C3	−3.8 (3)	C5—S1—C11—C12	−156.9 (3)
C1—N1—C3—C2	−3.9 (3)	C14—N2—C12—C11	165.7 (6)
C6—N1—C3—C2	−140.9 (3)	C13—N2—C12—C11	15.0 (4)
C1—N1—C3—C4	120.6 (3)	C13—C11—C12—N2	−14.4 (4)
C6—N1—C3—C4	−16.3 (4)	S1—C11—C12—N2	100.7 (4)
C7—C2—C3—N1	−116.8 (4)	C14—N2—C13—C11	−164.9 (6)
C1—C2—C3—N1	3.7 (3)	C12—N2—C13—C11	−15.1 (4)
C7—C2—C3—C4	136.5 (4)	C12—C11—C13—N2	14.3 (4)
C1—C2—C3—C4	−103.1 (4)	S1—C11—C13—N2	−93.9 (4)
N1—C3—C4—C9	−98.4 (5)	C15—N3—C14—N2	172.4 (7)
C2—C3—C4—C9	−0.1 (6)	C15—N3—C14—S2	−0.9 (7)
N1—C3—C4—C5	20.2 (4)	C12—N2—C14—N3	21.4 (9)
C2—C3—C4—C5	118.5 (4)	C13—N2—C14—N3	161.4 (5)
C9—C4—C5—C6	104.1 (4)	C12—N2—C14—S2	−165.3 (4)
C3—C4—C5—C6	−18.6 (4)	C13—N2—C14—S2	−25.3 (8)
C9—C4—C5—S1	−71.2 (5)	C16—S2—C14—N3	−12.7 (5)
C3—C4—C5—S1	166.1 (3)	C16—S2—C14—N2	173.4 (5)
C11—S1—C5—C6	156.3 (3)	C14—N3—C15—C16	18.3 (10)
C11—S1—C5—C4	−29.1 (4)	N3—C15—C16—S2	−26.1 (10)
C4—C5—C6—N1	9.5 (4)	C14—S2—C16—C15	21.0 (7)
S1—C5—C6—N1	−175.3 (3)	C18—O5—C17—O4	−80.2 (6)
C4—C5—C6—C10	−171.7 (4)	C10—O4—C17—O5	143.8 (4)
S1—C5—C6—C10	3.5 (5)	C17—O5—C18—O6	−0.7 (9)
C1—N1—C6—C5	−108.0 (4)	C17—O5—C18—C19	−179.7 (5)
C3—N1—C6—C5	4.8 (4)	O6—C18—C19—C21	−21.0 (12)
C1—N1—C6—C10	73.2 (5)	O5—C18—C19—C21	158.0 (9)
C3—N1—C6—C10	−174.1 (3)	O6—C18—C19—C20	−148.0 (9)
C1—C2—C7—O2	−50.0 (5)	O5—C18—C19—C20	31.0 (10)
C3—C2—C7—O2	51.5 (5)	O6—C18—C19—C22	98.0 (9)
C1—C2—C7—C8	−172.6 (4)	O5—C18—C19—C22	−83.0 (8)
C3—C2—C7—C8	−71.1 (5)		

### Hydrogen-bond geometry (Å, º)

**Table:**

*D*—H···*A*	*D*—H	H···*A*	*D*···*A*	*D*—H···*A*
O2—H2*A*···N3^i^	0.82	2.01	2.816 (6)	169
C11—H11*A*···O2^ii^	0.98	2.43	3.366 (6)	160

Symmetry codes: (i) *x*−1, *y*+1, *z*; (ii) *x*, *y*−1, *z*.
