# Supplementary material for: Crystal structure of tebipenem pivoxil
Source: Acta Crystallogr E Crystallogr Commun. 2018 Aug 10;74(Pt 9):1215–7. doi: 10.1107/S2056989018010770 (PMC6127708; doi:10.1107/S2056989018010770)
Supplement: Supplementary file 3 [file e-74-01215-sup3.pdf]

Supporting Information

Crystal Structure of Tebipenem Pivoxil

Chao Tang, Li Cai, Shuai Liu, Zhiwei Zheng, Gen Li, Jianli Chen, and Qiang Sui

Corresponding author email address: CAILI@mailbox.sc.edu (L.C.); chem_sq@163.com (Q.S.);

1. Synthesis and characterization of tebipenem pivoxil.

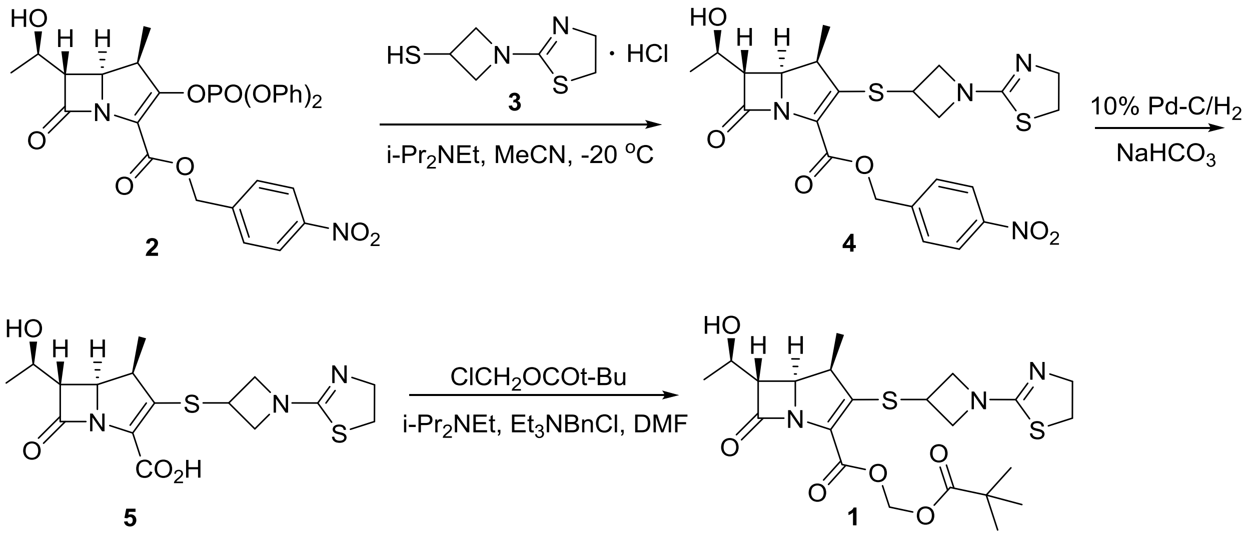

Table S1. Synthetic conditions for large-scale preparation of tebipenem pivoxil **1**.

Compound **4** reaction conditions					
batch #	core **2**	side chain **3**	molar ratio	yield %	m.p. (°C)
1	25.7 g, 43.2 mmol	10 g, 47.5 mmol	1:1.1	95	160 dec.
2	8.0 g, 13.5 mmol	3.1 g, 14.7 mmol	1:1.1	96	160 dec.
3	139.4 g, 234.5 mmol	54.3 g, 257.7 mmol	1:1.1	96	160 dec.
Compound **5** reaction conditions					
batch #	PNB ester **4**	7% Pd-C	molar ratio	yield %	m.p. (°C)
1	1.4 g, 2.7 mmol	0.83 g		58	186 dec.
2	5.1 g, 9.8 mmol	3.3 g		65	186 dec.
3	70 g, 135.0 mmol	41.7 g		58	186 dec.
Compound **1** reaction conditions					
batch #	compt. **5** tetrahydrate	chloromethyl pivalate	molar ratio	yield %	m.p. (°C)
1	1.9 g, 4.2 mmol	1.3 g, 8.6 mmol	1:2.05	77	134-136
2	28.6 g, 62.8 mmol	19.4 g, 128.8 mmol	1:2.05	77	134-136

2. ^{1}H NMR, ^{13}C NMR, and IR spectra of tebipenem pivoxil 1:

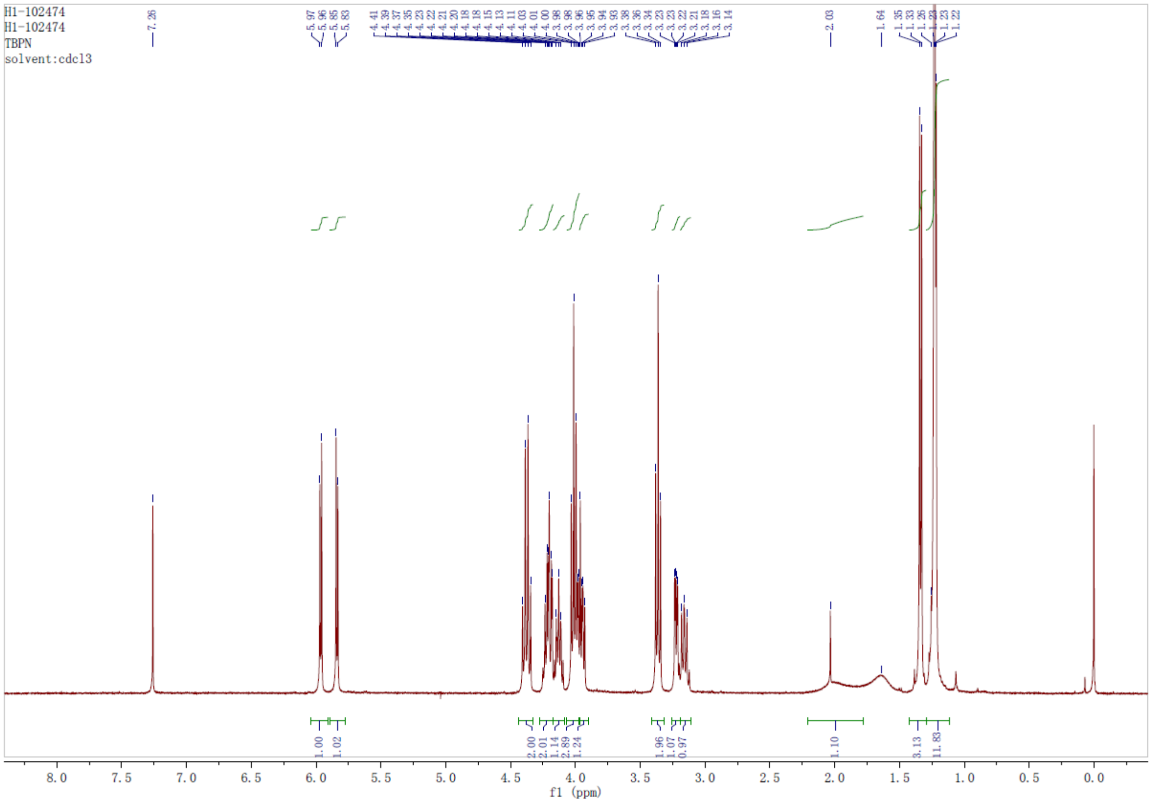
H1-102474
H1-102474
TBPN
solvent:cdcl3
f1 (ppm)

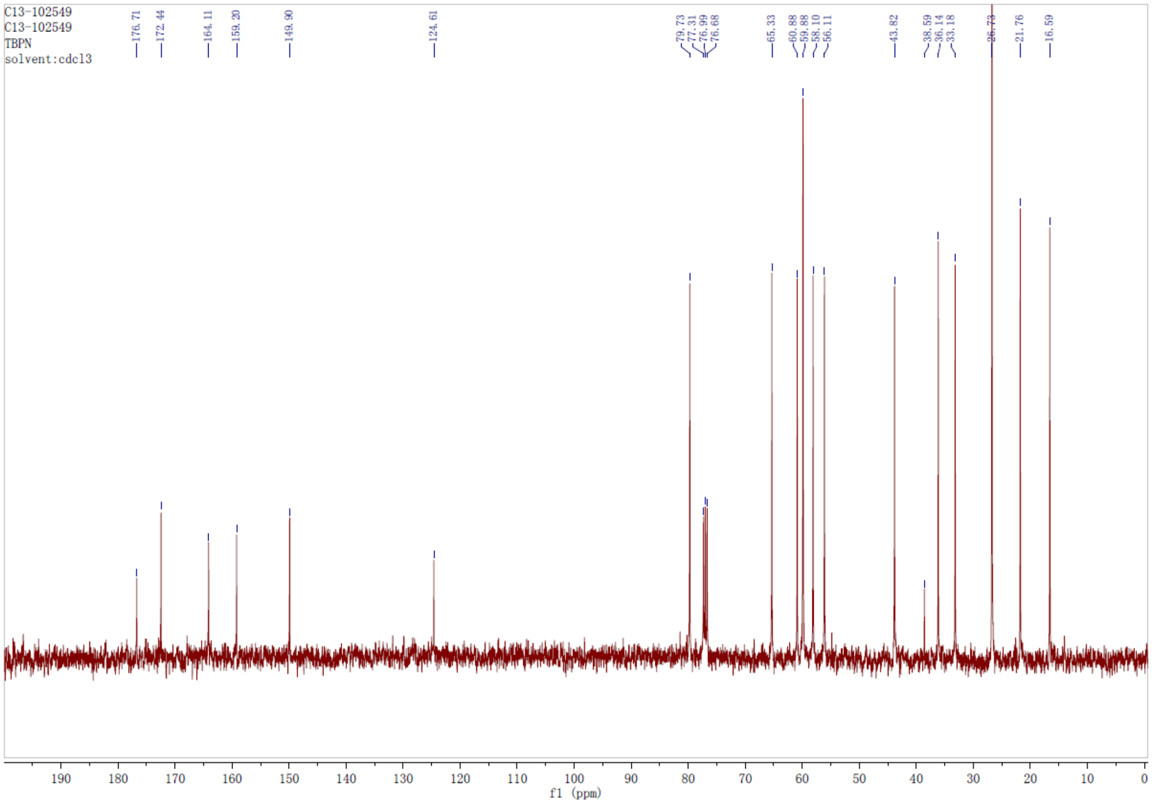
C13-102549
C13-102549
TBPN
solvent:cdcl3
f1 (ppm)

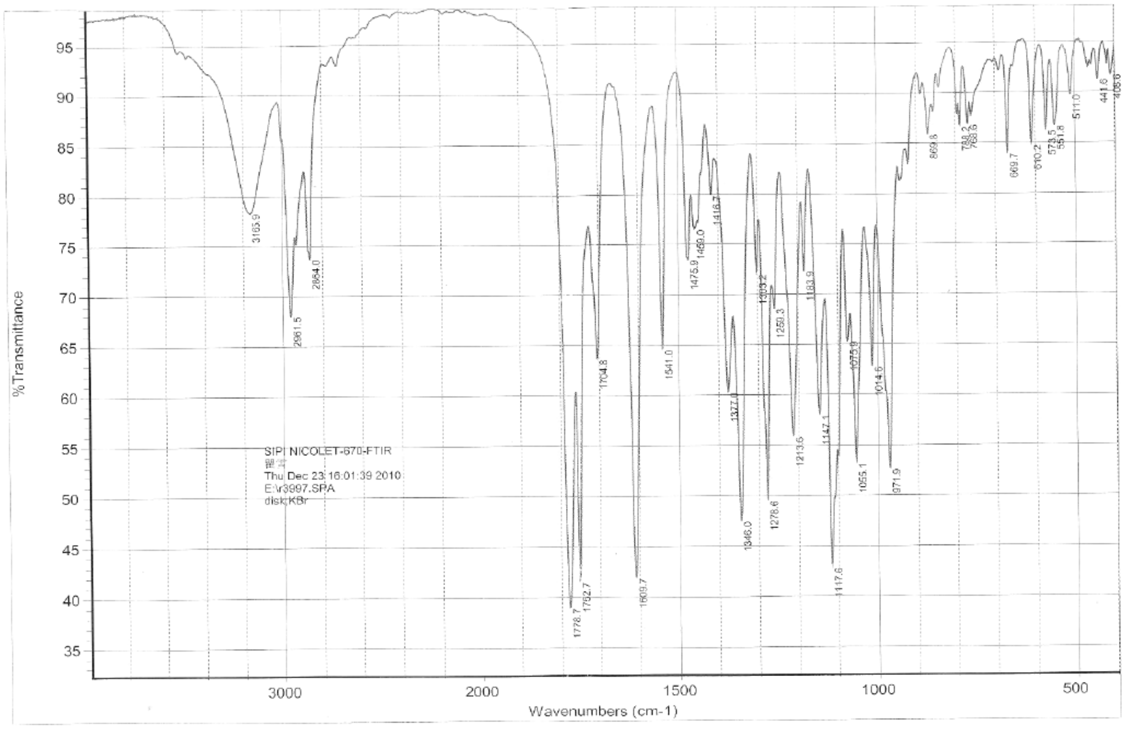
%Transmittance
Wavenumbers (cm-1)
SIPI NICOLET-670-FTIR
Thu Dec 23 16:01:39 2010
E:\r3997.SPA
disk:KBr
3163.9
2961.5
2854.0
1778.7
1752.7
1704.8
1609.7
1541.0
1475.9
1460.0
1416.7
1377.0
1346.0
1303.2
1278.6
1259.3
1213.6
1183.9
1147.1
1117.6
1075.9
1055.1
1014.6
971.9
869.8
788.2
768.5
669.7
610.2
573.5
551.8
511.0
441.6
406.6

Table S2. Selected bond lengths (Å) and bond angles (°) for tebipenem pivoxil **1**.

**Bond**	**Dist.**	**Bond**	**Dist.**	**Bond**	**Dist.**
S(1)-C(5)	1.734(4)	N(1)-C(6)	1.411(5)	C(4)-C(9)	1.525(6)
S(1)-C(11)	1.799(4)	N(1)-C(1)	1.413(5)	C(4)-C(5)	1.531(5)
S(2)-C(14)	1.779(4)	N(1)-C(3)	1.478(4)	C(5)-C(6)	1.343(5)
S(2)-C(16)	1.812(7)	N(2)-C(14)	1.315(6)	C(6)-C(10)	1.471(5)
O(1)-C(1)	1.194(5)	N(2)-C(12)	1.475(6)	C(7)-C(8)	1.513(5)
O(2)-C(7)	1.409(5)	N(2)-C(13)	1.477(6)	C(11)-C(13)	1.544(6)
O(3)-C(10)	1.197(5)	N(3)-C(14)	1.259(6)	C(11)-C(12)	1.548(5)
O(4)-C(10)	1.353(5)	N(3)-C(15)	1.484(6)	C(15)-C(16)	1.475(10)
O(4)-C(17)	1.432(5)	C(1)-C(2)	1.530(5)	C(18)-C(19)	1.522(7)
O(5)-C(18)	1.332(6)	C(2)-C(7)	1.499(6)	C(19)-C(21)	1.480(9)
O(5)-C(17)	1.371(6)	C(2)-C(3)	1.552(4)	C(19)-C(22)	1.498(12)
O(6)-C(18)	1.192(6)	C(3)-C(4)	1.553(5)	C(19)-C(20)	1.498(13)
**Angle**	**(°)**	**Angle**	**(°)**	**Angle**	**(°)**
C(5)-S(1)-C(11)	102.8(2)	N(1)-C(3)-C(2)	88.5(2)	O(4)-C(10)-C(6)	111.8(3)
C(14)-S(2)-C(16)	88.8(2)	N(1)-C(3)-C(4)	104.6(3)	C(13)-C(11)-C(12)	87.3(3)
C(10)-O(4)-C(17)	116.1(4)	C(2)-C(3)-C(4)	123.6(3)	C(13)-C(11)-S(1)	114.7(2)
C(18)-O(5)-C(17)	119.9(4)	C(9)-C(4)-C(5)	110.1(4)	C(12)-C(11)-S(1)	107.7(3)
C(6)-N(1)-C(1)	132.4(3)	C(9)-C(4)-C(3)	115.9(3)	N(2)-C(12)-C(11)	88.1(3)
C(6)-N(1)-C(3)	108.6(3)	C(5)-C(4)-C(3)	100.8(3)	N(2)-C(13)-C(11)	88.1(3)
C(1)-N(1)-C(3)	93.3(3)	C(6)-C(5)-C(4)	110.3(3)	N(3)-C(14)-N(2)	125.0(4)
C(14)-N(2)-C(12)	128.6(4)	C(6)-C(5)-S(1)	125.8(3)	N(3)-C(14)-S(2)	117.4(3)
C(14)-N(2)-C(13)	130.6(4)	C(4)-C(5)-S(1)	123.7(2)	N(2)-C(14)-S(2)	117.3(3)
C(12)-N(2)-C(13)	92.6(3)	C(5)-C(6)-N(1)	111.2(3)	C(16)-C(15)-N(3)	111.2(5)
C(14)-N(3)-C(15)	111.2(4)	C(5)-C(6)-C(10)	124.8(4)	C(15)-C(16)-S(2)	105.2(4)
O(1)-C(1)-N(1)	131.7(4)	N(1)-C(6)-C(10)	124.0(3)	O(5)-C(17)-O(4)	107.8(4)
O(1)-C(1)-C(2)	136.5(4)	O(2)-C(7)-C(2)	108.4(3)	O(6)-C(18)-O(5)	120.9(5)
N(1)-C(1)-C(2)	91.8(3)	O(2)-C(7)-C(8)	111.1(3)	O(6)-C(18)-C(19)	126.6(5)
C(7)-C(2)-C(1)	118.7(3)	C(2)-C(7)-C(8)	111.2(3)	O(5)-C(18)-C(19)	112.5(4)
C(7)-C(2)-C(3)	117.8(3)	O(3)-C(10)-O(4)	123.9(3)		
C(1)-C(2)-C(3)	86.0(3)	O(3)-C(10)-C(6)	124.2(3)		
